# Shape and weight concern as a moderator of program outcomes from the Prevention Across the Spectrum RCT

**DOI:** 10.1186/2050-2974-3-S1-O28

**Published:** 2015-11-23

**Authors:** Simon Wilksch, Tracey Wade, Susan Paxton, Susan Byrne, S Bryn Austin

**Affiliations:** 1 School of Psychology, Flinders University, Australia; 2 School of Psychological Science, La Trobe University, Australia; 3 School of Psychology, University of Western Australia, Australia; 4 Boston Children's Hospital, Australia; 5 Harvard Medical School & Harvard School of Public Health, Australia

## Objective

To investigate if baseline shape and weight concern moderated outcomes in the Prevention Across the Spectrum trial, a randomised-controlled trial (RCT) of 3 school-based programs aimed at reducing eating disorder and obesity risk factors.

## Method

N = 1,316 Grade 7 and 8 girls and boys (M age = 13.21 years) across three Australian states were randomly allocated to: Media Smart; Life Smart; Helping, Encouraging, Listening and Protecting Peers Initiative (HELPP) or control (usual school class). Risk factors were measured at baseline, post-program (5-weeks later), and 6- and 12-month follow-up.

## Results

Moderation was indicated by significant interaction effects for group (Media Smart; Life Smart; HELPP; Control) X moderator (high shape and weight concern; low shape and weight concern) X time (post-program; 6-month follow-up; 12-month follow-up), with baseline entered as a covariate. Such effects were found for shape concern, weight concern, eating concern, regular eating, body dissatisfaction, and physical activity. Post-hoc testing found Media Smart participants with high baseline shape and weight concern experienced a reduction in risk at 12-month follow-up for 4 of the 6 variables.

## Discussion

This study shows it is possible for a school-based program to reduce eating disorder risk factors in participants with high baseline risk of an eating disorder.

